# Overexpression of SLC38A1 is associated with poorer prognosis in Chinese patients with gastric cancer

**DOI:** 10.1186/1471-230X-14-70

**Published:** 2014-04-09

**Authors:** Jing Xie, Ping Li, Hui-feng Gao, Jian-xin Qian, Ling-Yan Yuan, Jie-jun Wang

**Affiliations:** 1 Department of Integrative Oncology, Fudan University Shanghai Cancer Center; Department of Oncology, Shanghai Medical College, Fudan University, 270 DongAn Road, Shanghai 200032, China; 2 Stem Cell Research Center, Renji Hospital, School of Medicine, Shanghai Jiaotong University, Shanghai 200127, China; 3 Department of oncology, Changzheng Hospital, the Second Military Medical University, 64 HeTian Road, Shanghai 200003, China

**Keywords:** Gastric cancer, Tissue microarray, Immunohistochemistry, SLC38A1, Prognostic factor

## Abstract

**Background:**

Current literature has demonstrated that host glutamine depletion facilitates tumorigenesis. Likewise, the glutamine transporter SLC38A1 is putatively associated with malignant transformation and tumor progression. Taken together, this forms the premise for undertaking the current study. The twofold aim of this study was to provide insight into whether or not a variance in the expression of SLC38A1 exists between human gastric cancer and healthy human tissues, and to determine how silencing the SLC38A1 gene could affect the proliferation, viability, migration, and invasion of gastric cancer cells.

**Methods:**

Immunohistochemical staining was used to analyze the expression of SLC38A1 in gastric cancer tissues and adjacent healthy mucosa in 896 patients with pathologically confirmed gastric cancer who had underwent R0 resection. SH-10-TC cells (a gastric cancer cell line) were used to examine whether silencing SLC38A1 with siRNA could affect cell viability, migration and invasion.

**Results:**

The SLC38A1 protein was very low or undetectable in healthy gastric mucosa. In contrast, strong staining of SLC38A1 protein was found in the cytoplasm in 495 out of the 896 gastric cancer samples. More pronounced SLC38A1 expression in gastric cancer tissues was significantly associated with age, differentiation status, lymph node metastasis, TNM stage and PCNA (proliferating cell nuclear antigen) expression. Upon univariate survival analysis, SLC38A1 expression was correlated with poor survival. Multivariate survival analysis revealed that SLC38A1 was an independent prognostic factor.

**Conclusion:**

SLC38A1 is overexpressed in gastric cancer, which suggests that it is contributory to tumor progression. These results encourage the exploration of SLC38A1 as a target for intervention in gastric cancer.

## Background

Amino acid transporters are essential for a variety of physiological cellular processes, including uptake of nutrients, energy and chemical metabolism, as well as malignant transformation [1]. Amino acid transporters include system A (ATA1/SNAT1/SLC38A1, ATA2/SNAT2/SLC38A2 and ATA3/SNAT4/SLC38A4), system L (LAT1/SLC7A5 and LAT2/SLC7A8), and y + (CAT2/SLC7A2). Among the various amino acid transporters, system L-type amino acid transporter-1 (LAT-1) is up-regulated in a wide variety of human cancers, including esophageal adenocarcinoma [2], oral squamous cell carcinoma [3], colorectal adenocarcinoma [4], and liver cancer [5]. System A amino acid transporter is also overexpressed in human cancers [5-8]. System A is a Na^+^-dependent active transport system, known to mediate the uptake of amino acids with small side chains (e.g., alanine, serine, proline and glutamine) [1]. Its activity is highly influenced by pH, cell volume, and a variety of hormones, such as insulin, glucagon, and insulin-like growth factor-I [1].

System A transporter in mammalian cells includes three distinct types, known as amino acid transporters SLC38A1, SLC38A2 and SLC38A4 [9]. These transporters belong to the solute-linked carrier family SLC38, and are differentially expressed among different organs/tissues. SLC38A1 is expressed primarily in the brain and placenta, as opposed to the lungs, liver, spleen and kidneys [10]. SLC38A2 is, on the other hand, ubiquitously expressed in mammalian tissues [9], while SLC38A4 is primarily expressed in the liver [11]. System A transporters are up-regulated in a range of human cancers, including breast cancer and hepatocellular cancer [6,7].

Our previous studies, using gene chip analysis, suggested elevated SLC38A1 mRNA expression in gastric cancer (unpublished data). In the current study, we compared the expression of SLC38A1 in gastric carcinoma in contrast with healthy adjacent gastric mucosa at the protein level. Potential correlation of SLC38A1 with the prognosis was examined using a multivariate analysis, and the biological role of SLC38A1 in proliferation and progression was examined in cultured gastric cancer cells using siRNA.

## Methods

### Patients

This study included 896 patients (median age: 61.4 years; 634 men, 262 women) with histologically confirmed gastric cancer who underwent D2/D3 curative resection at either Changhai or Changzheng Hospital in Shanghai, during a period from 2001 to 2005. Subjects who received neo-adjuvant therapy prior to the surgery were not included. Subjects with stage III disease also received a 5-fluorouracil-based chemotherapeutic regimen for 4–6 cycles. Patients presenting with neuroendocrine tumors, lymphoma or sarcoma were not included. Adjacent mucosa that was free from cancer cells was used as a healthy control.

The follow-up was conducted via phone conversation and mail in March 2010. Information with regards to survival/death was obtained in 673 cases (median survival: 59.08 months).

Demographic information and the construction of TMA blocks were described previously [12-15]. All tissue specimens were obtained with informed consent, and the use of the human specimens was approved by Institutional Review Board at Changhai and Changzheng Hospitals.

### Immunohistochemistry (IHC)

Immunohistochemical staining was carried out on 4-μm paraffin sections after heat-mediated antigen retrieval. Samples were incubated with a human-anti-rabbit polyclonal antibody to SLC38A1 (dilution 1:200; Abcam, Cambridge, U K) overnight at 4°C. Goat anti-rabbit antibody (dilution 1:4000; Invitrogen, Carlsbad, CA, USA) was used as a secondary antibody. An immunoglobulin-negative control was used to rule out non-specific binding. As for the positive control, we referred to other studies in liver cells by Nobuo Kondoh [6] and in hilar cholangiocarcinoma by Yu WL [16], which indicated the SLC38A1 protein was stained brown and diffused in the cytoplasm. Slides were counterstained with hematoxylin.

Data (staining in cytoplasma) were acquired independently by two investigators (Guan Zhen Yu and Ying Chen) blinded to sample nature using an Olympus CX31 microscope (Olympus, Center Valley, PA, USA), and analyzed using a semi-quantitative scoring system. Staining was graded on a scale of 0–2 (0 = negative staining [no cytoplasmic staining of any tumor cells], 1 = moderate expression [cytoplasmic staining of <25% of tumor cells], and 2 = high expression [cytoplasmic staining of ≥25% of tumor cells]) as described previously [14,17].

### Cell culture and siRNA transfection

SH-10-TC cells were obtained from Shanghai Institute for Biological Sciences, Chinese Academy of Sciences (CAS), and grown in RPMI-1640 medium containing 10% FBS. Cells were maintained at 37°C in a humidified atmosphere containing 5% CO_2_. The experiments were carried out in the exponential phase of growth.

Four pairs of anti-SLC38A1 siRNAs and randomized cocktails of dsRNA as a control were synthesized by GenePharma (Shanghai, China). The siRNA transfection was carried out using Lipofectamine 2000 (Invitrogen, Carlsbad, CA, USA).

### Western blot analysis

Cell lysate containing 100 μg protein was resolved on SDS-PAGE, transferred onto PVDF membrane and incubated with an anti-SLC38A1 antibody (dilution, 1:500; Invitrogen, Carlsbad, CA, USA). Protein bands were evaluated using an Odyssey infrared fluorescent scanning system (LI-COR Biosciences, Lincoln, NE, USA) and Scion Image software (NIH, USA). GAPDH was used as an internal control.

### Cell viability

SH-10-TC cells were seeded in 96-well plates at the day before transfection. Cells were counted at 48 h, 72 h and 96 h after transfection using a microtiter-plate colorimetric WST assay (Cell counting kit-8; Dojindo Laboratories, Kumamoto, Japan) at 450 nm. Experiments were conducted in triplicate.

### Transwell migration and invasion assay

Assays were performed using a modified Boyden chamber (Corning Costar, Rochester, NY, USA) containing a gelatin-coated polycarbonate membrane filter (8-μm pore size). The upper surface of the filter was coated with 20 μL Matrigel (0.3 mg/ml; BD Biosciences, Bedford, MA, USA). Transfected cells were harvested with a cell dissociation solution (Invitrogen, Carlsbad, CA, USA) and suspended in medium with 1% bovine serum albumin.

Cells (1 × 10^4^) were added to the upper compartment and allowed to migrate for 6 h at 37°C. After 6 h, cells on the upper side of the membrane were removed with a cotton swab. Cells on the bottom surface of the membrane were fixed in 3.7% paraformaldehyde, stained with hematoxylin for quantification. Data were expressed as the average number of cells per insert.

### Statistical analysis

The relationship between SLC38A1 expression and clinicopathologic parameters was analyzed using the chi-square test. Survival was analyzed using the Kaplan-Meier method and the log-rank test. The prognostic value of clinicopathologic parameters was examined using a Cox proportional hazards regression model for multivariate analysis. All tests were two-sided. *P* < 0.05 was considered statistically significant. All statistical analyses were performed using the SPSS statistical software program for Microsoft Windows (SPSS Inc., Chicago, IL, USA).

## Results

### SLC38A1 protein in gastric cancer

**Figure 1:**
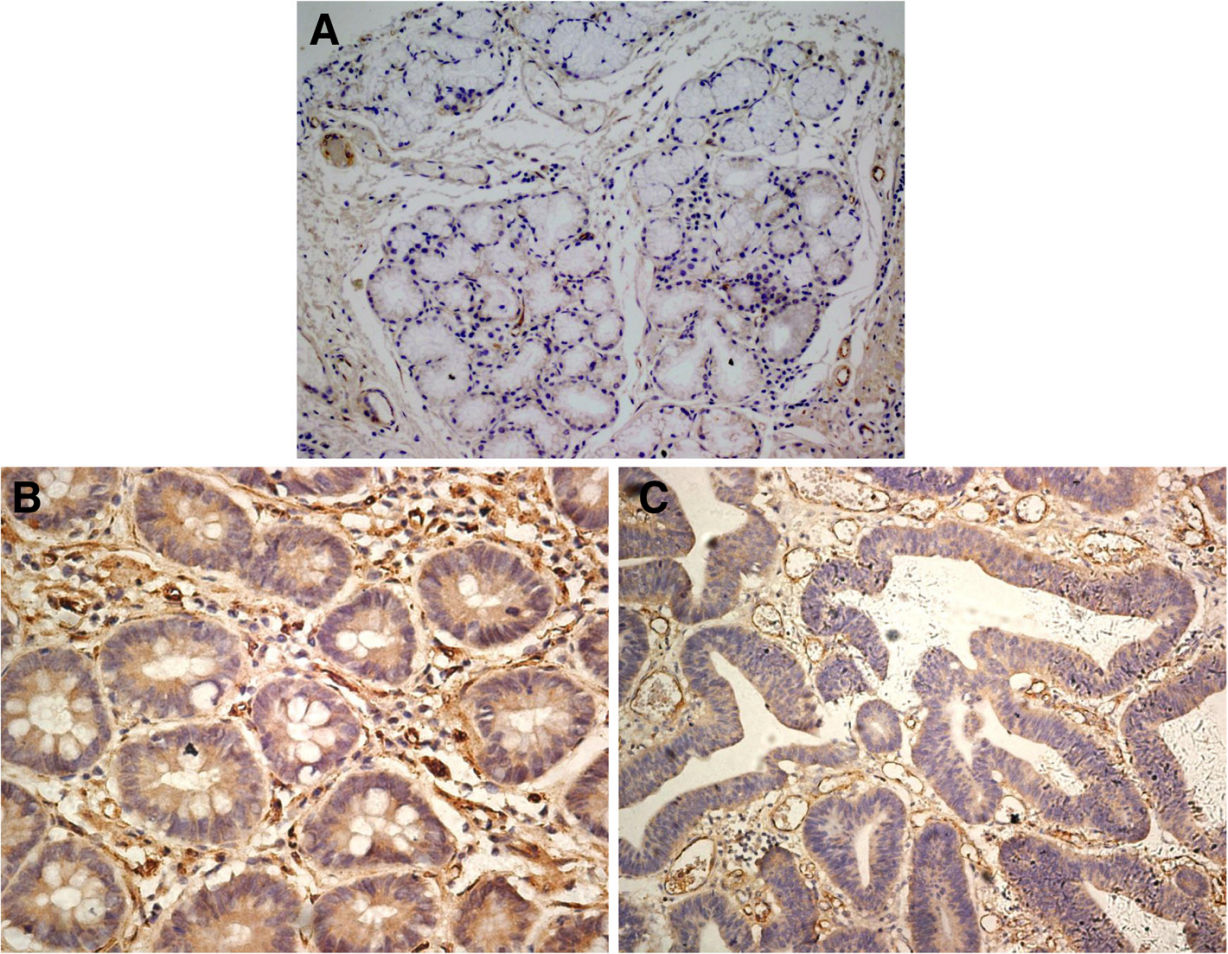
**Human gastric cancers and healthy mucosa specimens in tissue microarray were stained with an anti-SLC38A1. (A)** Representative healthy gastric tissue without SLC38A1 cytoplasmic expression, **(B)** Well-differentiated and **(C)** Morderately-differentiated gastric cancer tissues with positive SLC38A1 expression which was cytoplasm stained brown and diffuse. Magnification × 200.

The expression of SLC38A1 protein in the healthy gastric mucosa was very low or undetectable (Figure 1A). Strong staining of SLC38A1 protein in the cytoplasm was found in 495 out of the 896 cancer samples in both the well differentiated (Figure 1B), and poorly differentiated cancer cells (Figure 1C). In the cytoplasm, the SLC38A1 protein was stained brown and diffused.

### SLC38A1 expression and clinicopathologic factors

SLC38A1 overexpression was closely associated with old age (>60 yrs), differentiation status (high/moderate), regional lymph node metastasis, TNM stage (III), and positive PCNA expression (Table 1), but not with gender, tumor size, tumor location, operation manner, lymphovascular invasion, depth of tumor invasion, and p53 expression.

**Table 1:** Association between SLC38A1 expression and clinicopathological factors of GC patients

**Clinicopathological variables**	**Total**	**SLC38A1 positive expression**	
	**n**	**n**	***P***
Age			0.007
≤ 60 years	447	227	
> 60 years	449	268	
Sex			0.437
Male	634	345	
Female	262	150	
Location			0.277
Cardia	144	86	
Corpus	281	150	
Antrum	440	246	
Whole	31	13	
Operation manner			0.590
Subtotal gastrectomy	400	217	
Total gastrectomy	496	278	
Size (Diameter)			0.321
≤ 6 cm	706	384	
> 6 cm	190	111	
Differentiation			<0.001
High/moderate	553	337	
Low/undifferentiated	343	158	
Gastric wall invasion			0.151
T1/T2	308	160	
T3/T4	588	335	
lymph node metastasis			0.026
Negative	346	175	
Positive	550	320	
TNM stage			0.001
I/II	427	212	
III	469	283	
Lymphovascular invasion			0.275
Negative	850	466	
Positive	46	29	
p53 expression			0.382
Negative	401	228	
Positive	495	267	
PCNA expression			0.012
Negative	99	43	
Positive	797	452	

### Prognostic factors of overall survival

**Figure 2:**
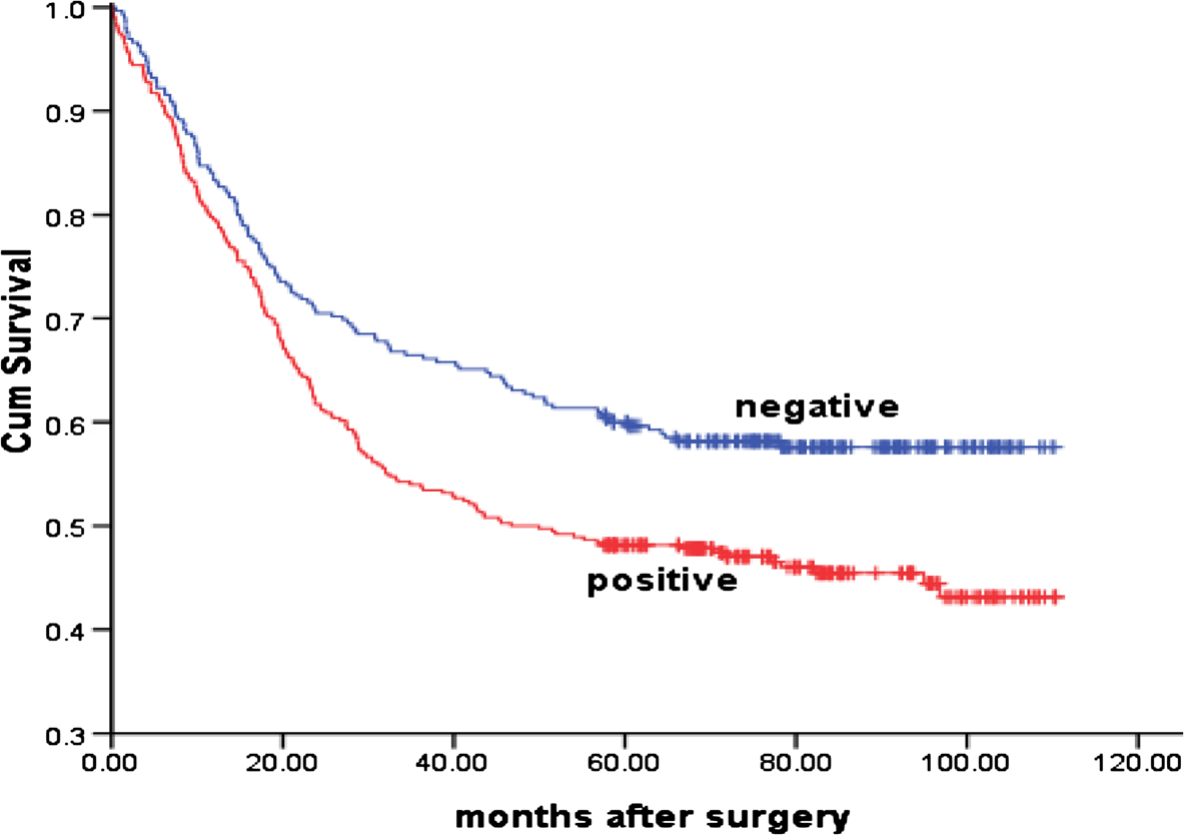
**Association between SLC38A1 expression and survival duration in patients with gastric cancer.** Kaplan-Meier survival analysis showed median survival of 46.69*vs.*69.7 months in patients with positive vs. negative SLC38A1 expression (*P* = 0.002).

SLC38A1 expression in cancer tissue was associated with poorer prognosis. The median survival was 46.69 months in patients with SLC38A1-positive cancers, and 69.7 months in subjects with SLC38A1-negative cancers (*P* = 0.002; Figure 2). Upon a multivariate analysis, independent prognostic factors for overall survival included SLC38A1 expression, differentiation status, lymphovascular invasion, PCNA expression, size and TNM stage (Table 2).

**Table 2:** Univariate and multivariate analysis of the prognostic significance of individual clinicopathological factors on survival

	**Univariate analysis**				**Multivariate analysis**			
	**HR**	**95.0% CI**		***P *value**	**HR**	**95.0% CI**		***P *value**
		**Lower**	**Upper**			**Lower**	**Upper**	
Size (≤ 6 cm vs. > 6 cm)	1.816	1.429	2.309	.000	1.327	1.039	1.696	.024
TNM (I/II vs. III)	4.214	3.292	5.393	.000	3.416	2.637	4.424	.000
Differentiation (high/moderate vs. low/undifferentiated)	1.876	1.509	2.332	.000	1.565	1.249	1.960	.000
Nerve invasion (negative vs. positive)	2.070	1.421	3.015	.000	1.353	.910	2.011	.135
Lymphovascular invasion (negative vs. positive)	2.664	1.770	4.011	.000	1.931	1.258	2.964	.003
PCNA (negative vs. positive)	1.794	1.174	2.742	0.007	1.695	1.098	2.617	.017
P53 (negative vs. positive)	1.277	1.024	1.593	0.03	1.046	.835	1.310	.695
SLC38A1 (negative vs. positive)	1.191	1.065	1.332	0.002	1.122	1.001	1.258	.041

### RNAi experiments

**Figure 3:**
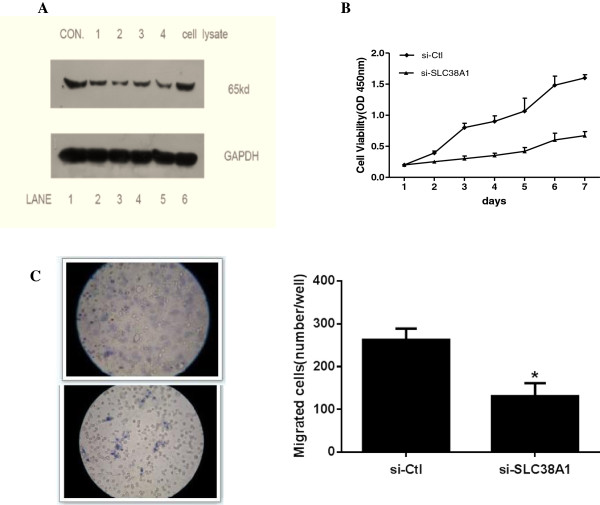
**Silencing SLC38A1 with siRNA in SH-10-TC cells and experiments in vitro. (A)** Representative Western blot in the siRNA experiments. siRNA #2 reduced the SLC38A1 transcript by >70% in comparison to scrambled control (lane1 and 6). GAPDH was used as the internal control. **(B)** CCK-8 assay shows si-SLC38A1 significantly inhibited the cell growth of SH-10-TC cells. **(C)** Upper panel: si-SLC38A1 decreased the migration of SH-10-TC cells in transwell chambers, compared to parental cells and si-Ctl group. Lower panel: migrated cells on the surface of the membrane (Columns, mean; bars, SD. *, *P* < 0.01 vs. scrambled control).

Specific knockdown of endogenous SLC38A1 protein was confirmed by all four siRNAs with Western blot (Figure 3A). siRNA #2 (si-SLC38A1) reduced the endogenous SLC38A1 transcript by >70%, and was selected for subsequent experiments.

Compared to parental cells and si-Ctl, si-SLC38A1 decreased the proliferation (Figure 3B), as well as the migration of SH-10-TC cells (Figure 3C), but did not affect cell passage through Matrigel.

## Discussion

Consistent with the physiological and pathological roles of glutamate in cancer development, we found marked differences in the expression of SLC38A1 in gastric cancer relative to adjacent non-cancerous tissue. The uncontrolled proliferation of neoplastic cells requires dramatic changes in energy metabolism [18], in which the primary metabolic fuel and nitrogen source for tumor cells in glutamine [19-21]. Glutamine is known to exert influence on a number of signaling pathways that contribute to tumor growth [22], and is additionally correlated with maintaining the TCA cycle and supporting NADPH production when glucose supply is limited [23].

While glutamine is not an essential amino acid, it plays an important role in maintaining the intestinal barrier structure and immune function. Within the three mammalian system A amino acid transporters, SLC38A1 has the highest affinity for glutamine, and is strongly expressed in glutamatergic neurons [24].

Low blood glutamine concentration has been found in patients with gastric carcinoma and conversely increased in the mucous membrane adjacent to the malignant tissue [25]. It is possible that ectopic activation of SLC38A1 may reflect a compensatory mechanism that confers a survival advantage.

### SLC38A1 expression in gastric cancer and other malignancies

In the present study, we found an increased expression of SLC38A1 in gastric carcinomas, relative to adjacent non-cancerous gastric mucosa, suggesting that SLC38A1 might play an important part in gastric cancer malignant transformation. Enhanced SLC38A1 expression has been observed in several other types of malignancies, including liver cancer [6], Hilar cholangiocarcinoma [16] and C6 glioma [26]. An elevated expression of SLC38A1 has been found to be closely linked to differentiation status, lymph node metastasis and TNM staging, and is thus implicated in the growth, invasion, metastasis and progression of gastric carcinomas.

### Potential mechanism of the action

Consistent with the observation in HepG2 liver cancer cell lines [6], our study showed that inhibiting SLC38A1 expression with siRNA decreased the growth of cultured SH-10-TC cells, thus indicating an overexpression of SLC38A1 contributes to oncogenesis of gastric cancer through promoting cell proliferation. Inhibiting SLC38A1 expression also reduced cell migration, providing evidence for increased cell migration as a mechanism for enhanced metastatic potential, and local invasiveness of SLC38A1-expressing tumor cells.

In breast cancer, scientists discovered that 17 beta-estradiol specifically increased System A activity by two to four-fold in estrogen receptor positive cell lines, with a maximum stimulation observed 48 h after estrogen-treatment. In estrogen receptor negative cell lines, however, no stimulation was observed, which provided evidence that estrogen receptors play a role in the activation of system A by estrogen [27]. In liver cancer, System A amino acid transporters produced a different kind of inactivation and substrate protection in membrane vesicles and reconstituted proteoliposomes, supporting the hypothesis that there were inherent differences presented in System A carriers in normal and transformed liver tissue [28]. In the current study, we found enhanced SCL38A1 expression in gastric cancer, which supported the implication of glutamine metabolism in tumors [29-31]. Interestingly, certain cancer cell lines are dependent on glutamine despite the fact that glutamine is a nonessential amino acid [32]. To be utilized by tumor cells, glutamine must be transported into tumor cell mitochondria. This implies that SLC38A1 could transport glutamine, and thus play an important role in neoplastic progression/development.

### Association of SLC38A1 expression with clinicopathological characteristics as well as prognosis in patients with GC

Upon further investigation of the relationship between SLC38A1 and clinicopathological factors, we found that overexpression of SLC38A1 was strongly associated with patient age, differentiation status, lymph node metastasis, TNM stage and the expression of PCNA, whereas no significant association was found in patient gender, tumor size, tumor location, operation manner, lymphovascular invasion, depth of tumor invasion, and p53 expression. These results indicated that SLC38A1 plays a central role in the malignant progression of gastric carcinomas.

Risk factors for gastric cancers have been reported in a number of studies, including differentiation, [33] nerve invasion [33], lymphovascular invasion [33], size [13,34], TNM stage [34], and some molecular markers [35]. Our study confirmed that differentiation, lymphovascular invasion and the TNM stage were independent predictors for gastric cancer. We additionally found that SLC38A1 expression alone was a prognostic factor, with its prognostic value in multivariate survival analysis for patients who underwent gastrectomy. Therefore, we supposed that overexpression of SLC38A1 is a significant factor associated with a poor prognosis, and might be a marker to forecast gastric cancer patients’ recurrence and survival. Secondly, patients with poorer prognosis should be monitored more closely and/or undergo more positive treatment.

## Conclusion

In conclusion, our study showed that SLC38A1 expression was closely associated with age, differentiation status, lymph node metastasis, TNM stage and PCNA expression, as well as a worse prognosis in patients with gastric cancer. The results from the experiments using cultured gastric cancer cells indicated that SLC38A1 expression could be used as an indicator of disease aggressiveness. Overall, these findings could be useful in developing a novel molecular target for individualized treatment of gastric cancer.

## Abbreviations

SLC38A1: Solute carrier family 38, member 1; TMA: Tissue microarray; PCNA: Proliferating cell nuclear antigen; TCA: Tricarboxylicacidcycle.

## Competing interests

The authors declared that they have no competing interests.

## Authors’ contributions

JX participated in the study design, collected patient material and drafted the manuscript. PL collected patient material, performed statistical analyses and revised the manuscript for important intellectual content. HFG participated in the study design and revised the manuscript for important intellectual content. JXQ collected patient material. LYY collected patient material. JJW conceived of the study, and participated in its design and coordination and revised the manuscript for important intellectual content. All authors read and approved the final manuscript.

## Authors’ information

JX, MD of oncology, was involved the application of National Natural Science Foundation associated with gastric cancer. Acquire the Foundation of collaboration with Comprehensive Integrative Medicine institute (CIMI), Republic of Korea. She is the secretary of Committee of Minimally Invasive Therapy in Oncology, Shanghai Anti-Cancer Association.

PL, MS of oncology, is involved the application of National Natural Science Foundation associated with gastric cancer. She is the recipient of a Doctor Innovation Foundation of the Medical of Shanghai Jiao Tong University.

HFG, MS of TCM, is involved the application of National Natural Science Foundation associated with pancreatic cancer. HFG has accumulated experiences in the follow-up and analysis the cases.

JXQ, MS of oncology, Vice Director, Department of Oncology, Changzheng Hospital, Shanghai, is involved the application of National Natural Science Foundation associated with gastric cancer.

LYY, MS of oncology, is involved the application of National Natural Science Foundation associated with gastric cancer.

JJW, Director, Department of Oncology, Changzheng Hospital, Shanghai. Director, Department of Oncology. He is the recipient of an award from the National Natural Science Foundation associated with gastric cancer. JJW has long been engaged in cancer research, especially gastric cancer.

## Pre-publication history

The pre-publication history for this paper can be accessed here:

http://www.biomedcentral.com/1471-230X/14/70/prepub
